# The feasibility of a dietary and lifestyle behaviour change intervention among low-income Latinas and Indigenous Mexicans in rural desert communities: a pilot randomised controlled trial

**DOI:** 10.1017/S1368980026102729

**Published:** 2026-06-01

**Authors:** Ann Marie Cheney, Jacqueline Moreira, Yameng Ge, Andrea Gonzalez, Michael Guzman Hernandez, Ashley Moran, Shaokui Ge

**Affiliations:** 1 Social Medicine Population and Public Health, University of California Riverside School of Medicine, USA; 2 Penn State, USA; 3 Johns Hopkins University Bloomberg School of Public Health, USA; 4 University of California Davis School of Medicine, USA; 5 Department of Research, University of California Riverside School of Medicine, USA

**Keywords:** Intervention, Latinx immigrants, Chronic disease, Indigenous Mexicans, Community health workers

## Abstract

**Objective:**

This article reports on the feasibility, acceptability of a trial design and results of ¡Coma, Muévase y Viva!, an adapted version of the Eat, Move, Live! Curriculum, a 10-week diet and lifestyle change intervention for low-income Latina and Indigenous Mexican women in rural Inland Southern California.

**Design:**

A pilot randomised intervention and wait-list controlled parallel trial.

**Settings:**

Latina and Indigenous Mexican women participated in ¡Coma, Muévase y Viva!, the adapted version of the curriculum customised for Spanish speakers with a focus on health literacy, cultural norms and values, and delivered by community health workers/promotoras.

**Participants:**

Participants were randomised into the intervention (*n* 19) or wait-list control (*n* 16). Most were Latino (88 %), mothers (75 %) and foreign-born (87 %).

**Results:**

The adapted intervention was feasible and acceptable with 87·5 % of participants retained at three months. The intervention was delivered with high fidelity. Intervention compared to control participants reported success with predetermined prioritised goals (OR = 0·25, 95 % CI: 0·06, 0·99, *P* = 0·03) and showed a greater likelihood of cooking healthier food (OR = 4·87, 95 % CI: 1·04, 22·90, *P* = 0·05), enjoyment of cooking healthy meals (OR = 3·94, 95 % CI: 0·87, 17·92, *P* = 0·08) and doing physical activities (OR = 5·30, 95 % CI: 1·16, 24·24, *P* = 0·03). Healthy eating and physical activity as primary outcomes indicated the intervention group was more likely to eat healthy and be active (OR = 2·08, 95 % CI: 1·12, 3·89, *P* = 0·02).

**Conclusion:**

The adapted intervention was feasible and acceptable and indicated an effect on dietary and lifestyle behaviour change with significant changes in meeting desired goals and engaging in healthy eating and physical activity.

The Latino/Hispanic population constitutes 19 % of the United States (US) population, projected to represent 28 % by 2026, representing the largest and fastest-growing minority group in the country^(1,2)^. This demographic shift is notable in rural areas, where the Latino population is the largest racial-ethnic group^(3)^. In both California and Texas, the two states with the highest percentage of Latinos/Hispanics in rural areas, 33 % comprise the rural demographic^(4)^. Among this demographic, Latinas (women with ties to Latin American countries) and Indigenous Mexican women whose Indigenous identity is rooted in communities and territories in Mexico rather than the US represent a significant and underserved subset, often experiencing chronic health conditions and comorbidities related to intersecting forms of marginalisation linked to gender, ethnicity, indigeneity and rural residence^(1,5)^.

This rapid demographic change presents significant public health concerns and challenges, especially for Latina and Indigenous Mexican women who face heightened health inequalities due to gender determinants of health^(6)^. This becomes prominently evident in the context of diabetes mortality, where death rates for Latina/Hispanic women are noticeably elevated (23·0 per 100 000) as compared to non-Hispanic White women (14·9 per 100 000)^(6)^. Compared to non-Hispanic Whites, Latinas are less likely to access diabetes prevention, management and control services, a gap that is even wider for Indigenous Mexican women, who often encounter additional challenges related to geographic isolation, cultural stigmatisation and structural inequities^(7)^.

Adherence to a healthy diet and lifestyle is critical to reduce risk for chronic disease burden^(8)^. Studies have demonstrated the impact of a healthy diet and lifestyle on risk reduction for chronic disease burden and have stressed the importance of intervention to mitigate premature disability and death^(9,10)^. For instance, a 12-month comparative effectiveness research trial found that a MyPlate-based intervention involving a cookbook that promoted fruit and vegetable choices, whole grains and replacing sugary beverages with water was highly effective in reducing weight and improving mental health and quality of life for low-income, urban Latinx and African American patients with overweight or obesity over time^(11,12)^. Yet, this study, like others, focused on urban populations. Research shows that rural environments can undermine intervention effects on weight loss presenting unique disadvantages such as limited access to supermarkets and exercise facilities to facilitate dietary and lifestyle change behaviours^(13)^.

The Latinx population in rural America faces distinct inequalities to realising dietary and lifestyle change behaviours^(14–16)^. Most are employed as low-wage agricultural labourers, making them the backbone of the American food system; yet, at the same time, Latinx agricultural labourers face food insecurity, lacking both adequate access and the financial means to purchase fresh produce and ingredients to prepare healthy meals^(17)^. This population needs access to evidence-based nutrition education and physical activity opportunities that considers their unique language, health literacy, cultural, socioeconomic and geographic needs^(18–20)^.

We build on this body of work and report on the cultural adaptation of the Eat, Move, Live! (EML!) programme, a community-based intervention to promote diet and lifestyle changes to decrease risk for cancer and chronic diseases by increasing knowledge, attitudes and behaviours related to nutrition, healthy eating and physical activity. EML! is considered an effective public health intervention with individual peer-review studies demonstrating positive effects on weight loss, reduction in BMI and waist-to-hip ratio, and changes in attitudes and beliefs around chronic diseases (from being irreversible to preventable via behaviour change) from baseline to 12-week follow-up^(21)^. The EML! curriculum, delivered in-person or virtual by bilingual (English and Spanish) staff, includes weekly nutrition education, food preparation and physical activity sessions. The original curriculum, intended for low-income racial/ethnic minority women, reduces barriers to healthy eating, but is delivered by professionals, requires advanced health literacy skills among programme participants and does not include social engagement opportunities important for intervention adherence^(21)^. As part of a pilot study, funded by the National Institutes of Health, we sought to culturally adapt the EML! intervention for low-income, Latinx and Indigenous Mexican women in rural farm-working communities in Southern California, many of whom face barriers to accessing health information due to language, educational and structural factors^(14,18)^. Prior to the beginning of the adaptation process, the study’s leadership met with the developers of the EML! curriculum and discussed adaptations for language (Spanish material), health literacy levels, and intervention delivery.

The purpose of this article is to report on the feasibility and acceptability of delivering an adapted version of the EML! intervention and its preliminary effects on dietary and lifestyle change. We also briefly describe the cultural adaptations of the EML! curriculum.

## Methods

### Study design and objectives

This study was carried out by Unidas por Salud, a community academic partnership team led by an academic investigator, a bilingual (English and Spanish) medical anthropologist and health services researcher and a community investigator, a bilingual (Spanish and Purépecha) advocate and public health researcher. Unidas por Salud builds the capacity of minority women to serve as community health workers (CHW)/promotoras, trusted community leaders, to partner in health disparities research. For this project, the academic and community investigator led a team of nine CHW/promotoras trained in human subjects research, recruitment, data collection and analysis, and clinical trial designs that was supported by eight student research assistants. The CHW/promotoras lived in the Eastern Coachella Valley where the study took place and were trusted leaders in their community. The research team was equipped to engage with the community since all team members were fluent in Spanish and two team members (community investigator and a CHW/promotora) were bilingual in Spanish and Purépecha, an indigenous language spoken by members of the Purépecha community from the Mexican state of Michoacán.

We conducted a prospective pilot randomised controlled (RCT) parallel trial using an allocation ratio of 1:1 or close between the intervention group and wait-list control design to assess feasibility and acceptability and test the potential trend of intervention effects of the adapted intervention for dietary and lifestyle changes. This study employed a community-based participatory research approach, a collaborative approach to research characterised by power sharing, shared decision-making and equitable resource allocation^(22)^. This approach was operationalised through active collaboration between the community investigator and team of CHW/promotoras, ensuring their involvement in all phases of the project.

This study included the following objectives: (1) evaluate the feasibility and acceptability of the intervention, (2) assess the potential intervention effects measured by a few correlated items measuring healthy lifestyles and (3) assess the potential changes in perceived chronic disease burden. This research was approved by the Institutional Review Board at the University of California, Riverside and participants provided their informed consent for research participation prior to the start of research activity. The study was funded by the National Institutes of Health-National Cancer Institute, Award #5P20CA242620-03 and registered with clinicaltrails.gov, National Institutes of Health National Library of Medicine.

### Setting

This research was carried out in the Eastern Coachella Valley, part of a rural desert region in Inland Southern California. This region is among the richest agricultural areas in the world and is home to a large Latinx and Indigenous Mexican immigrant population who live and work in the nearby agricultural fields. Despite harvesting crops for the American food system, this population is food insecure and experiences chronic disease burden related to diet and lifestyle factors as well as limited access to preventive care and healthcare services^(18,23,24)^. Foods harvested in the Eastern Coachella Valley by this Latinx farm-working population are shipped to wealthier markets making it difficult for farm workers to access and afford locally grown produce. Many in the region access the regional food bank, a chapter of Feeding America, which provides food assistance to food insecure individuals and families in the region.

### Intervention

This project involved tailoring the EML! programme, an existing lifestyle and dietary behaviour change intervention developed by City of Hope for health literacy, language and cultural relevance^(25)^. We followed the five-step cultural adaptation process described by Barrera *et al.* ^(26)^ and used information gathered from our previous intervention research on diabetes and diet and lifestyle change to modify the existing intervention material for language, health literacy and food access^(18)^.

The original intervention programme, EML!, is a dietary and lifestyle behaviour change intervention focused on reducing risk for cancer and chronic disease burden. The intervention is an in-person or virtual 10-week programme that includes weekly sessions. Each session is designed to be 120 min and includes a 60-minute presentation on a health topic, a 30-minute cooking demonstration on a healthy recipe and a 30-minute exercise activity. The health topics include the following: (1) MyPlate dietary guidelines, (2) sugary drinks, (3) nutrition labels, (4) obesity, (5) diabetes, (6) stress, (7) cholesterol and heart disease, 8) eating healthy with limited economic resources, (9) body image and mental health and (10) reducing food waste. Recipes vary and include plant-based meals with grains such as pastas and quinoa and use of spices to augment taste. Exercise classes include yoga, band exercises and aerobic activity. The material for participants is in both English and Spanish; however, the manual for intervention implementation is in English only. Additionally, the required literacy level is high: intended for an audience with at least a high school degree if not some college.

### Adaptation

Our adaptation of the intervention, referred to in Spanish as ¡Coma, Muévase y Viva!, was collaborative engaging CHW/promotoras in the process and involved both surface- and deep-level changes^(27–29)^. Surface-level changes also referred to as ‘presentation strategies’ may include bilingual and bicultural material, ethnic foods and images, delivery methods (e.g. by CHW, group settings) and culturally appropriate activities; whereas, deep-level changes or ‘content strategies’ incorporate cultural values into the design and/or delivery and implementation of the intervention^(26,29)^. As a first step, we translated the existing intervention implementation manual from English to Spanish. The manual provides instructions on how to use the programme’s material and engage participants in the intervention. Next, the academic team used the Spanish language manual to tailor the existing material (i.e. PowerPoint slides) for health literacy level, language and cultural relevance. The community academic team, including the academic and community principal investigators, CHW and student research assistants, collaborated on adaptations to the PowerPoints and handouts. Surface-level modifications included the following: (1) developing all PowerPoints and handouts in Spanish, (2) reducing text and incorporating ethnic and culturally relevant images, descriptions and videos to convey key components of health education topics and (3) using images and colours representative of Latinx immigrant families and communities with Mexican heritage (e.g. vibrant colours). Deep-level changes involved the following: (1) incorporating culturally specific illnesses and explanatory models (e.g. *susto* [fright] and diabetes) into health education material, (2) using culturally relevant recipes aligned with Latin American foodways and (3) including exercise activities (Zumba) and equipment accessible to low-income Latinx communities (e.g. water bottles as weights, and Latin American music to motivate participants to move). Additionally, sessions were held in the evening so that women working in the fields could join after work.

Another deep-level change, per input from our team of CHW/promotoras, was the addition of a WhatsApp group to the intervention to disseminate weekly recipes and exercises and create a forum for group conversation and social engagement. We chose WhatsApp, as it is a commonly used platform in the community. CHW/promotoras helped participants unfamiliar with the application install it and provided instruction on how to use it. Recipes included MyPlate-based recipes from our team’s Ancestral Recipes cookbook, which includes recipes aligned with Mesoamerican food traditions^(18).^ An exercise plan prepared by a CHW/promotora was posted that included instruction on the type of exercise and duration as well as videos demonstrating the recommended exercises. A weekly prompt to spark conversation was also posted. Prior to the start of the intervention, a study team member placed all participants randomly assigned to the intervention arm into the WhatsApp group.

Instructional material from the Spanish language manual was incorporated into the notes section of PowerPoint slides to facilitate fidelity to intervention delivery. Nine CHW/promotoras were trained to deliver the intervention virtually via Zoom video conferencing in a group setting and encourage engagement with messages shared via WhatsApp that were posted by a research assistant. Participants were instructed to download the Zoom application and enter the meeting from their home; as such, all assigned participants were present in the Zoom space and participated from their own homes. The CHW/promotoras, who were well-versed in the Zoom platform, provided support and instruction to participants on how to download the Zoom application to their smartphone and how to click on the link to access the virtual session. For some participants, the CHW/promotoras also helped with downloading the WhatsApp application and navigating the group chat.

### Sample size estimation

It was expected that the intervention would have the standardised effect between small and medium sizes; thus, the sample size per group was set up between fifteen and twenty-five for 90 % power and two sided tests^(30)^.

### Data collection

Data collection was carried out from July 2022 to December 2022. Trained CHW/promotoras administered pretest and post-test surveys in-person or via Zoom or phone. Pretest surveys were collected one week prior to the start of the intervention and post-test surveys within one week of the close of the intervention; both took approximately 45–60 min to complete. BMI and health status were also assessed at baseline and follow-up. For baseline measures, participants attended a free clinic where trained team members obtained height and weight for all participants and assessed health status using a GRIPX Digital hand dynamometer, a tool used to measure hand grip strength, at baseline and follow-up^(31)^. Research has shown that grip strength is a valid and reliable measure of a variety of health conditions, including cancer and CVD^(31,32)^.

For follow-up measures, trained CHW/promotoras went to participants’ homes to obtain these same measures. Additionally, the team collaborated with a clinical partner to collect blood samples at baseline and follow-up to conduct HbA1c tests, a measure of blood glucose over three months. Participants’ blood was drawn at baseline, and HbA1c levels were obtained. However, due to ethical concerns, the community academic team chose not to collect follow-up blood samples and HbA1c levels were not used in the analysis. For baseline and follow-up data collection, participants received a gift card after the completion of the in-person BMI and health indicator (fitness grip) measurements: $75 for baseline data collection (pretest survey, BMI, hand grip strength, blood sample) and $75 for follow-up data collection (post-test survey, BMI, hand grip strength).

### Randomisation

A total of forty participants were recruited into the study. Two study research assistants randomly assigned participants to one of the two arms: twenty intervention and twenty wait-list control. Participant identification numbers were input into an online group randomisation tool (https://www.randomlists.com/team-generator), which randomly allocated study participants to either the intervention or wait-list control arm. Nineteen participants in the intervention arm and sixteen in the control arm completed follow-up data collection. Sample completeness was assessed by comparing the distribution of sociodemographic characteristics between the two arms.

Participants in the wait-list control arm did not receive the intervention during the study observation period; post-test survey, body measurements and fitness measurements were collected from both study arms prior to delivery of the intervention to wait-list participants. However, at the start of the study, prior to randomisation, all participants received a cookbook with culturally relevant and healthy recipes including those assigned to the wait-list control.

### Feasibility

Participant retention and intervention fidelity were used to assess feasibility of implementing the intervention with the target population. Retention was measured using participant tracking logs to document attendance and reasons for discontinuation with the study. We considered the study feasible if 80 % of participants were retained in the intervention for three months. Intervention fidelity was measured using in vivo observations and video recordings of intervention implementation and participant enjoyment and satisfaction survey questions. The National Institutes of Health Behavior Change Consortium framework for individual-level behaviour change via intervention guided fidelity assessments for the study design, including CHW training, intervention receipt and enactment^(33)^.

#### Fidelity to study design

To measure alignment of the study design with the original hypothesis that programme participation would result in lifestyle and dietary behaviour change, we assessed dosage, including duration and frequency of the intervention components (health education, physical activity, cooking demonstration), and CHW/promotora adherence to intervention delivery through in vivo observations during intervention implementation and by reviewing the video recordings of each session. The CHW/promotoras attended all sessions, including sessions they did not lead, for ongoing training purposes.

#### Fidelity to training on CHWs/promotoras

CHW/promotoras completed a minimum of 25 h of training including an initial workshop on the EML! programme for its three components (education, physical activity and cooking) and the goals of the programme. The training was followed by a training with a medical doctor on nutrition and chronic disease prevention. CHW/promotoras also participated in weekly sessions to review the health education material, preparation and cooking of recipes and physical activity exercises, prepare for implementation and discuss any challenges or concerns with intervention delivery.

#### Fidelity to intervention delivery

The community academic team reviewed the delivery of the three components on a weekly basis during team meetings. Deviations from the intervention delivery model were discussed and corrected. Additionally, team members were present during the live sessions and reviewed video recordings of the sessions to ensure key components of the intervention (health education, physical activity and cooking demonstration) were completed.

#### Fidelity to intervention receipt

To measure participants’ understanding of and engagement with information shared during the intervention sessions, study team members and CHW/promotoras held a debriefing after each session. CHW/promotoras indicated their perceptions of participants’ level of engagement in session activities (e.g. questions posed, activity level, interest in educational material) and understanding of the material. Research assistants also reviewed participants’ responses to prompts shared in the WhatsApp group chat to assess understanding of health education and skill development (e.g. cooking healthy recipes and physical activity). Participants were prompted to share either via text or videos their cooking of healthy recipes or physical activities.

#### Fidelity to intervention enactment

To assess the use of intervention skills for lifestyle and dietary change behaviours, we assessed lifestyle change goals and incorporation of healthy meals and physical activity into daily routines. For the goals, participants provided free-text responses regarding their three goals by priority for the programme prior to the start of the intervention. Text responses were analysed to reduce items into themes or patterns across participant responses and coded as follows: eating healthy, maintaining weight, preventing chronic health outcomes, physical activity, cooking healthy, healthy family habits and motivation. At the end of the intervention period, participants were asked whether they had completed their prioritised goals, were planning to complete them or had no plans yet to complete them. Post-intervention delivery survey questions assessed whether participants cooked healthy foods (yes/no), defined as minimally processed foods (e.g. fresh fruits and vegetables, grains *v*. less natural foods with additives and artificial ingredients), and the weekly frequency of cooking healthier meals.

### Acceptability

To measure acceptability, we assessed, for the intervention arm participants only, their enjoyment of cooking healthy recipes and doing physical activity (yes/no) demonstrated during classes.

### Intervention effects: healthy lifestyle and chronic disease burden

To assess the potential effects of the adapted intervention on health outcomes for a future trial, we collected baseline and follow-up data on healthy lifestyle behaviours and perceived burden of chronic disease.

#### Healthy lifestyle

Healthy lifestyle was measured by assessing healthy eating and physical activity using a 4-point Likert scale. The following questions assessed healthy eating: (1) ‘On average, how many times per week do you and your family eat out (including take out eaten at work or home, or meals at a fast food restaurant, carryout or drive-through)?’ with response options of none, 1–2, 3–5, more than 6 times per week; (2) ‘How many servings of vegetables do you eat every day?’ with response options of none, 1–2 servings, 3–4 servings, 5–9 servings; (3) ‘On average, how many glasses of water do you drink per day?’ with response options of none, 1–2, 3–4, 5–7, 8 or more; (4) ‘In the past week, did you drink any soda/sugary drinks?’ with response options of none, 1, 2–3, 4–6, 7 or more times; and (5) ‘In the past week, did you eat any sweets/junk food?’ with response options of none, 1, 2–3, 4–6, 7 or more. Participants were provided with examples of sweets and junk food such as soda, chips, candy, cookies, French fries, etc. The following questions assessed physical activity: ‘How many days per week do you do moderate physical activity for at least 30 min (e.g. brisk 1–2 d/week walking, sports, Zumba)?’ with response options of 0, 1–2 d/week, 3–4 d/week, 5 or more days/week.

The responses to these questions captured different aspects of a healthy lifestyle and were therefore treated as correlated outcomes. A linear mixed model was used for analysis. Although such responses are often combined into a single summed score – implicitly assuming that each item contributes equally to the overall measure – this assumption is likely unrealistic. The proposed linear mixed model accounted for the correlation among the responses while still assuming equal contribution of each item to the overall construct.

#### Perceived chronic disease burden

Participants’ perceptions of chronic disease burden were assessed using the following question: ‘Please tell us how these comorbidities of overweight/obesity are a problem for you or your family’ with response options on a three-point Likert scale from not at all to a great deal. Comorbid conditions included diabetes, high blood pressure, heart disease/cholesterol/stroke, cancer, asthma/respiratory illnesses and being overweight.

### Socio-demographics and covariates

The demographic characteristics were collected at enrolment into the programme, including age, racial/ethnic heritage, country of origin, primary language, English proficiency, educational attainment, employment status, marital status, family size, number of children, household income and health insurance. Food insecurity was measured using the six-item US Household Food Security Survey (https://www.ers.usda.gov/topics/food-nutrition-assistance/food-security-in-the-us/survey-tools) as well as by asking about accessing food dispensary sites: ‘In the last month, how many times have you been to a dispensary to pick up food?’ General concerns about chronic health conditions (e.g. overweight, diabetes) were also collected. Additionally, BMI was calculated by collecting height and weight and used the National Heart, Lung and Blood Institute BMI calculator and categories: underweight < 18·5; normal weight 18·5 – 24·9; overweight 25–29·9 and obesity = 30 or greater (https://www.nhlbi.nih.gov/health/educational/lose_wt/BMI/bmi-m.htm).

### Data analysis

#### Sample summary

The sample was summarised and compared across two groups. Continuous variables were described using the mean, median and range (minimum and maximum), and group differences were assessed using the Wilcoxon rank-sum test. Categorical variables were presented as frequencies and percentages, and the group differences were evaluated using Fisher’s exact test.

#### Recruitment effort, eligibility rate and retention rate

Recruitment effort was measured by the number of women contacted and the amount of time spent on outreach. The recruitment success rate was calculated as the percentage of successfully enrolled participants out of the total contacts made. The eligibility rate reflected the percentage of women who met the study criteria and agreed to participate. Retention rate was defined as the percentage of enrolled women who completed the programme and returned all required surveys. Women were eligible if they: (1) were 18 years of age or older, (2) identified as Latina or Indigenous Mexican (e.g. Purépecha), (3) lived in the rural desert region of Inland Southern California and (4) were of low income.

#### Feasibility and acceptability

Feasibility was assessed through fidelity to five key components: (1) training of CHW/promotoras (measured by time spent and sessions completed), (2) adherence to the intervention design, (3) fidelity in intervention delivery, (4) participant receipt of the intervention and (5) participant enactment of intervention strategies. Fidelities 1–4 were quantitatively described based on time invested and the specific content areas addressed. Fidelity to intervention enactment was evaluated using frequencies and percentages derived from participants responses to two key questions: (1) whether participants had achieved the first three healthy lifestyle goals they set and (2) whether participants had implemented healthy cooking practices into their cooking routines. These responses were further analysed using logistic regression for binary outcomes (e.g. yes/no) and ordinal mixed-effects regression models (i.e. cumulative link model) for ordinal outcomes (e.g. frequency categories)^(34,35)^. In these models, the categorised responses served as dependent variables, while group assignment was the independent variable. Models were adjusted for demographic covariates, and results were reported as adjusted OR with 95 % CI and associated *P*-values.

Acceptability was assessed for intervention group participants only and was based on participants’ enjoyment of preparing healthy foods and engaging in physical activity as introduced in the intervention classes. Responses were summarised using frequencies and percentages. To examine differences in the degree of enjoyment, responses were categorised as ‘not at all,’ ‘a bit’ and ‘a lot’ and analysed using ordinal regions, consistent with the approach described above.

In addition, Cronbach’s Alpha tested the reliability of constructed items for the survey constructs of healthy lifestyle and perceived chronic disease burden outcome variables, which provided statistical parameters to determine if and how the constructs performed individually and differently with the assumption they would be highly correlated to measure different aspects of the proposed measure for the future RCT.

## Results

**Figure 1.**
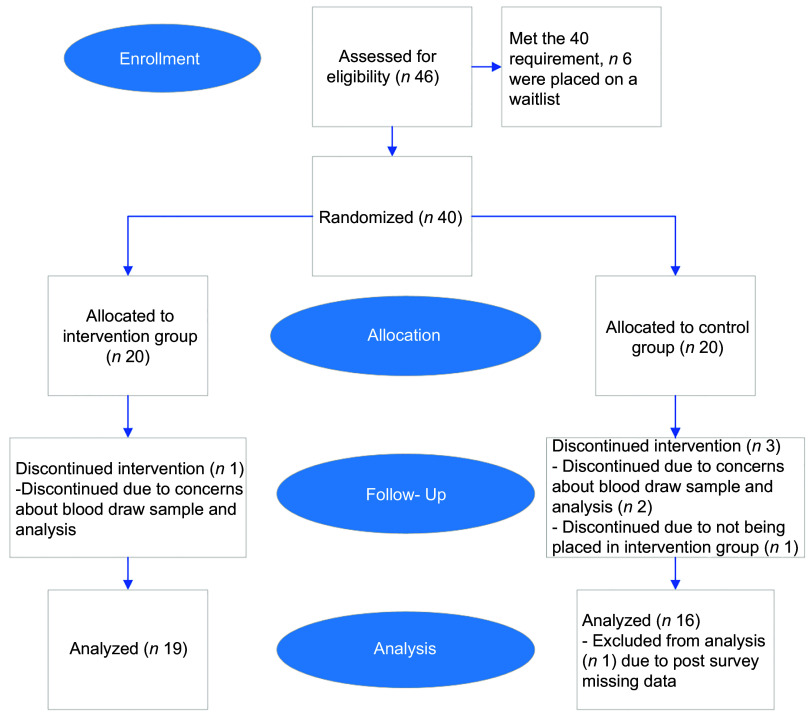
Participant recruitment and retention.

The goal was to recruit an initial sample of forty women with the intent to have a final sample of 30 (75 %) women to account for an anticipated 25 % attrition. A total of forty-six women were recruited, of whom six (13·0 %) were either not eligible or opted out of study participation. Forty were randomised to either the intervention arm or wait-list control arm of whom thirty-five (87·5 %) were retained and completed the post-intervention survey (see Figure 1). Participant attrition occurred at the beginning of the study. Some participants withdrew after completion of baseline blood sample collection. Others opted out due to logistical considerations related to randomisation, including assignment to the wait-list control group.

### Feasibility

#### Fidelity to training

CHW/promotoras retained knowledge and skills obtained during training sessions throughout intervention delivery. Weekly meetings and debriefing sessions permitted the CHW/promotoras to discuss their experiences, express concerns and obtain feedback from team members conducting in vivo observations. In total, CHW/promotoras conducted ten sessions over ten consecutive weeks, ensuring that all randomised participants received the same standardised information.

#### Fidelity to study design

CHW/promotoras implemented the same dose to all participants who received an average of 95 min per session with an average of 57 min of health education, 27 min of physical activity and 11 min of cooking demonstration.

#### Fidelity to intervention delivery

In vivo observations and review of video recordings demonstrate high fidelity to intervention delivery. Team members attended all ten sessions and monitored all WhatsApp posts. Across all ten sessions, the CHW/promotoras included the key components of the intervention (health education, physical activity, cooking demonstration) and the weekly post was published to WhatsApp within 2 d following each virtual session. A key component with low adherence was the cooking demonstrations that were designed to be 30 min, including a 15-minute demonstration followed by a discussion of the benefits of healthy eating and ways to incorporate healthy eating into daily cooking routines.

#### Fidelity to intervention receipt

Observations and review of videos and WhatsApp posts by participants in the intervention arm indicate high engagement with information and recommended behaviour changes. Participants consistently asked questions during health education presentations and participated in both the cooking demonstrations and exercises. Over the 10 weeks of the intervention period, participants published a total of 158 posts to the WhatsApp group chat with eight related to health education, 122 of cooking healthy recipes and twenty-eight of physical activity. Health education posts included the sharing of health resources and comments about health. Cooking posts included pictures of foods or meals cooked, comments about having cooked something healthy or comments on others’ healthy foods/meals. Physical activity included pictures documenting exercise (e.g. total number of steps in a day), videos of exercising and comments about physical activity such as ‘I walked today.’ While participants were not asked to record their daily steps as part of study participation, several participants used their smart device to measure their daily steps on their own and shared this information with others in the WhatsApp group chat.

#### Fidelity to intervention enactment

Across the intervention and control groups, healthy eating, physical activity and cooking healthy meals were the top three goals. Table 1 shows significant differences between the two groups with regards to achieving prioritised goals at the follow-up assessment. Only one person in the intervention group compared to five in the control group had not yet planned to achieve their top prioritised goal. While not statistically significant, there were between-group differences in reaching the second or third goals with 84 % of participants in the intervention group having completed or planned to complete their second goal and 72 % their third goal and 75 % in the control group having completed or planned to complete their second goal and 75 % their third goal. When building an ordinal mixed model using ‘yes’ as the control, it was shown OR = 0·25 (95 % CI: 0·06, 0·99, *P* = 0·03), implying that, compared to the control, the intervention group was less likely to respond with ‘not planned yet’ over ‘is planning’ or ‘yes.’

**Table 1.** Assessment of fidelity and acceptability of the adapted intervention

	Control group						Intervention group						*P* value
	*n*	%	*n*	%	*n*	%	*n*	%	*n*	%	*n*	%	
Enactment of goals achieved at follow-up													
	Completed		Planned		No plan		Completed		Planned		No plan		
Goal 1	3	19	8	50	5	31	7	39	10	56	1	6	0·03
Goal 2	3	19	9	56	4	25	4	21	12	63	3	16	0·89
Goal 3	4	25	8	50	4	25	4	22	9	50	5	28	0·99
	*n*			%			*n*			%			
Enactment of knowledge and skills about cooking healthy food at follow-up													
1. Cooking healthier food													
Yes	10			62			5			26			0·04
No	6			38			14			74			
2. Weekly frequency of cooking healthier food													
0 times	10			67			5			28			0·05
1–2 times	3			20			5			28			
>= 3 times more	2			13			8			44			
Acceptability of intervention curriculum													
1. Enjoyed preparing healthy meals demonstrated in classes													
Not at all							1			5			
A little							8			42			
A lot							10			53			
2. Enjoyed doing physical activities demonstrated in classes													
Not at all							1			5			
A little							7			37			
A lot							11			58			

Furthermore, participants in the intervention group were more likely to cook healthy foods multiple times per week compared to those in the control group indicating only 1–2 times per week. In mixed linear model-based inferences, the intervention group was significantly more likely to cook healthier food (OR = 4·87, 95 % CI: 1·04, 22·90, *P* = 0·05).

#### Acceptability

The intervention group reported enjoyment of preparing healthy food and doing the physical activities shared during classes (see Table 1).

### Intervention effects on healthy lifestyle and chronic disease

The pilot RCT included thirty-five adult female participants (*n* 35), with an average age of 44 years (sd = 12). Most (90%) identified as Hispanic, with Mexico (77%) or Central America (6%) as their place of origin, and 8·6 % identifying as Indigenous Mexican Purépecha. Spanish was the most common (74%) primary language used at home. While about half (51%) reported obtaining an education below the high school level, the majority (95%) reported a ‘normal’ reading proficiency. Over two-thirds were married, nearly half (49%) reported a family size of five or greater and 63% had at least 2 children. Over half (54%) earned an annual household income below $25 000. Sixty percent had health insurance and 43% depended on a government-funded health programme (Table 2). Over half regularly accessed a food dispensary, and 62% were food insecure (low to very low food security) as assessed by the US Household six-item food security screening tool. All participants were at least overweight with a BMI exceeding twenty-five. Nearly three-fourths were classified as having obesity.

**Table 2.** Demographic characteristics for control and intervention groups

	Control (*n* 16)		Intervention (*n* 19)		*P* value
	*n*	%	*n*	%	
Age					
Mean	43		44		0·71
sd	8·1		15		
Median (Min, Max)	41	30, 60	43	21, 67	
Ethnic group					
Latino	14	88	17	89	0·78
Indigenous Mexican	2	12	1	5	
Caucasian	0	0	1	5	
Household size					
Mean	4·7		4·0		0·27
sd	1·8		1·7		
Having children					
Yes	12	75	10	53	0·41
No	4	25	8	42	
Marriage					
Single	2	12	3	16	0·77
Married or living with partner	14	88	16	84	
Education					
Below High school	9	56	9	47	0·99
High school	3	19	4	21	
More than High school	4	25	5	26	
Household income					
< 15K	2	12	5	26	0·77
15–25K	6	38	6	32	
25K–40K	5	31	6	32	
>= 40K	3	19	2	11	
Health insurance					
Employee plan	4	25	2	11	0·60
Governmental plan	6	38	9	47	
None	6	38	8	42	
BMI					
Overweight	5	31	4	22	0·70
Obesity	11	69	14	78	

### Intervention effects – healthy lifestyle and chronic disease

Intervention effects for the primary outcome are shown in Table 3. Despite being a feasibility trial and not powered to detect statistical significance between the intervention and control group, those in the intervention compared to the control group were significantly more likely to eat healthy and be active (OR = 2·08, 95 % CI: 1·12, 3·89, *P* = 0·02). Those in the intervention group were twice as likely to consume daily fruit, vegetables and water and significantly reduce fast food consumption.

**Table 3.** Assessment of the intervention effects on healthier lifestyle and perceived chronic disease burden using mixed ordinal regression models*

The main outcomes of models for group comparisons: intervention *v*. Control group	Adjusted OR	95 % CI	*P* value
Model 1: Healthy lifestyle	2·08	1·12, 3·89	0·02
Model 2: Perceived chronic disease burden	6·09	1·31, 35·46	0·03

*Two mixed ordinal regression models were used, with each outcome specified as an ordinal dependent variable. The independent variable in both models was study group (intervention *v*. control). Each model was adjusted for baseline responses to the corresponding survey questions on lifestyle behaviours and perceived chronic disease burden, respectively. The models were not adjusted for demographic covariates because the two groups were demographically homogeneous; both consisted of ethnically similar Hispanic/Latina women with comparable ages, and Spanish was the primary language spoken.

For perceived chronic disease burden, the changes in percentages of participants reporting ‘not at all’ from various conditions were calculated from pre- to post-test. As shown in Table 3, the intervention group showed improvement in all conditions, with an increase in percentages of ‘not at all’ responses on most conditions except cancer (OR = 6·09, 95 % CI: 1·31, 35·46, *P* = 0·03). The mean summed score difference in changes from pre- to post-intervention showed no significant difference.

## Discussion

The goal of this pilot study was to assess the feasibility, acceptability and preliminary efficacy of a group-based, virtual dietary and lifestyle change intervention for low-income Latinas in rural, farm-working communities in Southern California. We found the intervention to be both feasible and acceptable. The adapted intervention was likely successful as it resonated with participants’ cultural norms and values regarding food and Latin American food ways and incorporated social engagement opportunities and personalised goal setting facilitating adherence to changes in diet and lifestyle^(8,18)^. It also had significant effects on healthy eating, water consumption and physical activity. These findings will inform the planning and development of a future definitive trial to be conducted with a larger sample sufficient to detect statistically significant differences among study arms for the main outcome variables.

The CHW/promotoras successfully recruited 40 participants into the study and retained 87·5 % of participants at 3 months. The engagement of these participants, who experience multiple intersecting marginalised identities (e.g. low-income, Latina, immigrant, indigenous) in our clinical trial, is laudable. Historically, racial-ethnic minority groups, rural residents and individuals and families of lower socioeconomic status are underrepresented in research generally and clinical trials specifically^(36,37)^. Mistrust of research and the scientific community, including concerns about potential harm, data misuse and limited familiarity with clinical research, remains a significant barrier to clinical trial participation among minoritised populations^(38)^. In this study, mistrust was most evident in relation to blood-based measures, particularly A1C testing. Several participants expressed discomfort or concern regarding blood collection. These concerns contributed to participant attrition following baseline data collection, as some participants withdrew from the study.

While Latinos/Hispanics remain substantially underrepresented in clinical trials, accounting for only 6-7 % of participants nationally, despite disproportionate risk for CVD, type II diabetes and cancer^(39,40)^, our findings underscore the promise of community-engaged approaches. Consistent with prior studies, the use of a model in which CHW/promotoras played a central role supported recruitment and participant engagement among Latinx and Indigenous Mexican community members, contributing to the overall feasibility of the study^(41)^.

The CHW/promotoras also delivered the intervention with high fidelity. All delivered each of the three intervention components (health education, cooking demonstration, physical activity) across the ten classes. Participants were engaged both during classes and the WhatsApp group chat posting about health resources, pictures of healthy foods cooked, videos of physical activity and posts on exercise success (e.g. total daily steps). Participants in the intervention group demonstrated their knowledge and skills learned by reporting healthy cooking and exercise into daily routines. Our study findings align with behavioural health intervention research demonstrating success with CHW/promotores delivering programme curriculum. For instance, the Entre Familia Reflejos de Salud study used a promotora model to deliver a home-visit healthy eating intervention among mothers of Mexican origin in southern California^(42)^. Mothers in the intervention group were much more likely to increase daily vegetable consumption, as well as make dietary changes (e.g. fibre-rich foods). This speaks to the importance of family-based interventions in Latinx families and the role of mothers in enacting dietary and lifestyle behaviour change within their family system^(43)^.

The use of digital health interventions for lifestyle modifications in rural, Latino communities is increasingly common and feasible. As our team, and others have found, engaging the focal community in the design and development of digital health interventions is critical for success, as is providing trainings and support as participants engage in the intervention^(44)^. The adapted intervention was likely feasible to implement and acceptable because we obtained input from CHW/promotoras and incorporated their insights on potential challenges to digital access and user-friendly platforms commonly accessed by community (e.g. WhatsApp). We noticed that digital access, especially the use of WhatsApp, was not as accessible for participants who indicated Purépecha as their preferred language. This may be due to illiteracy as many Purépecha-speaking immigrants in the US had limited formal education in Mexico and may not have learned Spanish formally limiting their reading and writing abilities. In such cases, our team orally translated from Spanish to Purépecha the WhatsApp dialogue recording it and then sharing it with these participants.

In addition to being both feasible and acceptable, the intervention was effective. While this pilot study was not powered to detect significant differences between the intervention and control arm for intervention effects, we did find meaningful changes in healthy eating (e.g. reductions in weekly sugar and soda consumption) post-study for the intervention group. Furthermore, intervention participants were much more likely to enjoy cooking healthy foods/meals and doing physical activity compared to the control group.

Low-income Latinos in rural, farm-working communities are often unable to consume healthy, nutrient-dense foods to reduce risk for chronic disease burden^(18,45)^. It is well established that food insecurity, which is high among Latinx farmworkers, increases the consumption of processed, high-carbohydrate and low-nutrient sugary foods, exacerbating chronic health conditions^(45–47)^. Over half of our sample was food insecure per the US Household six-item food security screening tool, and most regularly accessed food dispensaries. Through deep-level changes to the original EML! Curriculum, the adapted intervention addressed food insecurity. Content changes to the material included inclusion of resources for access to local dispensaries and shared ways to use ingredients and food items commonly distributed by dispensaries to prepare healthy meals. CHW/promotoras demonstrated MyPlate-based recipes featured in our Ancestral Recipes cookbook, that was designed in collaboration with the local chapter of Feeding America and uses low-cost and easily accessible ingredients^(18)^.

Furthermore, Latinos in rural America struggle to meet guidelines for recommended physical activity due to a lack of recreational facilities (e.g. gyms), sidewalks for walking or running, green spaces and public transportation to access facilities^(19,48)^. Domogalla *et al.* ^(19)^ found that cultural factors and familial obligations often take precedence over exercise, which further reduce opportunities for physical activity. This digital health intervention offered exercise activity that could be done within 20–30 min and within the home. Participants in our study lived in environmental justice communities where safety concerns and exposure to environmental hazards, such dust storms, agricultural chemicals and sulphuric smells emitted from the Salton Sea, pollute the air and are harmful to those who breathe it deterring outdoor activities for adults and children^(23)^. The physical activity exercises in the intervention include indoor activities focused on the use of calisthenics (e.g. lunges, squats), aerobics (e.g. marching in place) and resistance training with light weights. These exercises do not require much space or workout gear or equipment. For instance, most participants did not own a set of dumbbells or hand-held weights in their homes. CHW/promotoras, many of whom also did not own such gear, encouraged use of water bottles in lieu of weights and placing a towel on the floor in place of a yoga mat. This approach offered a solution addressing the challenges low-income populations experience to making dietary and lifestyle behaviour changes.

### Limitations

Several limitations should be considered in the interpretation of study findings. First, while the fidelity to intervention delivery for the health education and physical activity was high, it was low for the cooking demonstration. Our review of the recorded sessions indicates that the CHW/promotoras followed the guidelines in terms of cooking the selected recipe and engaging participants in a discussion about healthy foods and lifestyle choices; yet the actual time spent demonstrating how to cook the recipe and discussing ways to lead healthy lives by healthier food choices were, on average, only 11 min. This was a third of the expected time spent on this intervention component. Cooking demonstrations are a time-consuming and difficult task. The person doing the demonstration must often practice prior to the ‘live’ session, prepare all ingredients beforehand and review any health-related material and simultaneously cook, share health education information and engage audience members in the process. Doing this all successfully requires training and practice. Based on our assessment of this study, we recommend assigning this task to CHW/promotoras who feel confident cooking in front of others and dividing the labour between two people: one focuses on the cooking and the other focuses on sharing health information. Both can engage audience members in the process. Another consideration could be shifting to a cooking class, rather than a demonstration, and holding the class in person. This way participants have hands-on practice with cooking healthy foods, which has been proven to be quite effective in healthy eating^(18)^. Such an approach would require providing participants with food ingredients and more intensive training for CHW/promotoras hosting the cooking class.

Second, there were several errors with our measurements. First, the items for the healthy lifestyle and perceived chronic disease burden were developed by the original EML! team and informed by validated tools (e.g. National Cancer Institute Diet History Questionnaire); yet they are not validated measures. This is problematic especially for questions requiring dietary recall (e.g. past week eating habits). Our team also made an error in our initial measurements of BMI, primarily due to the scale being placed on the carpet. This oversight went unnoticed until about halfway through data collection. Consequently, the CHW/promotoras scheduled an in-home data collection visit with these participants within one week of baseline data collection to re-measure their weight. While we anticipated a decrease in the BMI of the intervention group, the results indicate a slight (but not statically significant) increase from baseline to follow-up. Thus, we surmise that the slight increase is likely because of the short intervention period (10 weeks) and small sample size.

Third, we were unable to use the HbA1c data as part of our primary outcomes. HbA1c, a crucial marker for monitoring diabetes management, could have provided valuable insights into the effectiveness of the intervention among participants with diabetes or at risk for diabetes. However, due to ethical concerns regarding the collection and preparation of participants’ blood samples, the community academic team in collaboration with the university’s Institutional Review Board, decided to stop all blood collection and remove the data from the analysis. When conducting research involving vulnerable populations, in our case a social and economically disadvantaged population that experience inequities in health due to citizenship, race/ethnicity, indigeneity and language, ethical considerations are of paramount importance^(14)^. Our team, like others conducting health disparities research with vulnerable populations, encountered ethical concerns regarding the collection, preparation, storage and analysis of participants’ biological samples^(49,50)^. We ultimately sought to protect participants in our study from exploitation and undue harm due to what we perceived as a lack of transparency, good clinical practice and professional ethics.

Last, as part of this pilot RCT, we estimated the intervention effects to assess their potential significance for informing a future trial. Measures of healthy eating and physical activity were treated as correlated outcomes to evaluate intervention effects. Because these outcomes were assessed using Likert scale items, they were initially analysed using summed scores – an approach that assumes each item contributes equally to the overall construct. However, this assumption may not hold in practice. A linear mixed model was used to account for the correlation among items, while an ordinal regression model, which is more appropriate for Likert-type data, was also considered^(51)^. Therefore, in the proposed test, we assessed the potential of the proposed outcomes to use for a future definitive trial. Given the relatively small sample size, formal statistical inference is generally not recommended due to limited power. Nevertheless, the observed effects in this analysis indicated sufficient statistical power, suggesting that the intervention has promising potential and supporting the rationale for conducting a fully powered trial.

### Conclusion

The adapted intervention was acceptable and feasible. Our team of CHW/promotoras recruited and retained a historically underrepresented group in a 3-month pilot RCT. Project success likely reflects our community-based participatory research approach involving shared leadership and decision-making, as well as the engagement of community leaders (i.e. CHW/promotoras) in all aspects of the research from the study design to intervention implementation and evaluation. Equalising power hierarchies and employing horizontal leadership, a collaborative approach emphasising shared decisions and responsibilities, is a best practice for the engagement of historically underrepresented populations in research^(52,53)^ By having CHW/promotoras from the community with expertise in health disparities research learn and implement the curriculum, our team overcame the common barriers to the engagement of minorities and underserved populations in social and behavioural trials: mistrust, cost and time commitment and trial accessibility and awareness^(37–39)^. Our community-based participatory research approach arguably contributes to increased representation of marginalised populations in scientific innovation and advancement in public health programming and healthcare delivery.

## Supporting information

10.1017/S1368980026102729.sm001 Cheney et al. supplementary material 1 Cheney et al. supplementary material

10.1017/S1368980026102729.sm002 Cheney et al. supplementary material 2 Cheney et al. supplementary material

## Supplementary material

For supplementary material accompanying this paper visit https://doi.org/10.1017/S1368980026102729
