# Supplementary material for: The feasibility of a dietary and lifestyle behaviour change intervention among low-income Latinas and Indigenous Mexicans in rural desert communities: a pilot randomised controlled trial
Source: Public Health Nutr. 2026 Jun 1;29(1):e148. doi: 10.1017/S1368980026102729 (PMC13434616; doi:10.1017/S1368980026102729)
Supplement: Supplementary file 1 [file S1368980026102729sup001.docx]

**Supplementary Table 1**. Healthy lifestyle outcomes across the two groups

| Healthy lifestyles measure for eating, drinking, and physical activities  *Cronbach Alpha, 0.63* | | | | |
| --- | --- | --- | --- | --- |
| Components of the lifestyle | Control Group  (n=16) | | Intervention Group   (n=19) | |
|  | Pre. Test mean (SD) | Post. Test mean (SD) | Pre. Test  mean (SD) | Post. Test mean (SD) |
| Daily fruit consumption | 2.1 (0.5) | 2.3 (0.5) | 2.3 (0.6) | 2.5 (0.5) |
| Daily vegetable consumption | 1.6(0.8) | 1.9 (0.7) | 1.9 (0.8) | 2.2 (0.9) |
| Fast food daily | 2.1 (0.3) | 2.3 (0.5) | 2.1 (0.6) | 2.3 (0.5) |
| Daily water intake | 2.1 (0.9) | 2.3 (0.8) | 2.4 (0.8) | 2.7 (0.6) |
| Weekly sugary food consumption | 2.3 (0.6) | 2.4 (0.7) | 2.1 (0.8) | 2.6 (0.7) |
| Weekly soda intake | 2.3 (0.7) | 2.4 (0.7) | 2.3 (0.6) | 2.7 (0.6) |
| Weekly moderate exercise | 1.6 (0.6) | 1.9 (0.9) | 1.9 (0.9) | 2.3 (0.9) |
| Summed scores | 14 (1.8) | 15 (2.6) | 16 (2.4) | 17 (2.7) |
