# Supplementary material for: The feasibility of a dietary and lifestyle behaviour change intervention among low-income Latinas and Indigenous Mexicans in rural desert communities: a pilot randomised controlled trial
Source: Public Health Nutr. 2026 Jun 1;29(1):e148. doi: 10.1017/S1368980026102729 (PMC13434616; doi:10.1017/S1368980026102729)
Supplement: Supplementary file 2 [file S1368980026102729sup002.docx]

**Supplementary Table 2**. Between-group differences in change in perceived concern about chronic health conditions

| Perceived chronic health conditions | the difference of the percentages changed of responses of not concern at all  *Cronbach Alpha, 0.67* | | |
| --- | --- | --- | --- |
|  | Control (n=16) | Intervention (n=19) | Difference |
| Diabetes | -18% | 24% | 42% |
| High blood pressure | -6% | 13% | 19% |
| Heart disease/cholesterol/stroke | 0% | 11% | 11% |
| Cancer | -18% | -10% | 8% |
| Asthma/respiratory illness | -25% | 21% | 46% |
| Overweight | 0% | 16% | 16% |
| Summed changes of % | -67% | 75% |  |
